# Probabilistic edge inference of gene networks with markov random field-based bayesian learning

**DOI:** 10.3389/fgene.2022.1034946

**Published:** 2022-11-10

**Authors:** Yu-Jyun Huang, Rajarshi Mukherjee, Chuhsing Kate Hsiao

**Affiliations:** ^1^ Division of Biostatistics and Data Science, Institute of Epidemiology and Preventive Medicine, National Taiwan University, Taipei, Taiwan; ^2^ Department of Biostatistics, Harvard University, Boston, MA, United States; ^3^ Bioinformatics and Biostatistics Core, Center of Genomic Medicine, National Taiwan University, Taipei, Taiwan

**Keywords:** Bayesian markov random field, edge prioritization, existence probability, gene regulatory network, network structure, probabilistic association

## Abstract

Current algorithms for gene regulatory network construction based on Gaussian graphical models focuses on the deterministic decision of whether an edge exists. Both the probabilistic inference of edge existence and the relative strength of edges are often overlooked, either because the computational algorithms cannot account for this uncertainty or because it is not straightforward in implementation. In this study, we combine the Bayesian Markov random field and the conditional autoregressive (CAR) model to tackle simultaneously these two tasks. The uncertainty of edge existence and the relative strength of edges can be measured and quantified based on a Bayesian model such as the CAR model and the spike-and-slab lasso prior. In addition, the strength of the edges can be utilized to prioritize the importance of the edges in a network graph. Simulations and a glioblastoma cancer study were carried out to assess the proposed model’s performance and to compare it with existing methods when a binary decision is of interest. The proposed approach shows stable performance and may provide novel structures with biological insights.

## 1 Introduction

The network analysis of multi-dimensional data for structural information learning has attracted much attention in the biomedical research community. Examples include gene regulatory networks, brain connectivity networks, and microbial networks (Zhang et al., 2019; Huang et al., 2020). An undirected graphical model, the Markov random field (MRF), is a common approach to describe the network structure of a group of genetic variables, because of its direct interpretation of edges with the conditional dependence between nodes. The Gaussian MRF, also known as the Gaussian graphical model (GGM), imposes a multivariate distribution for gene regulatory networks, assuming the p -dimensional vector $X={\left(X_{1},X_{2},\ldots X_{p}\right)}^{T}\in R^{p}$ follows a multivariate normal distribution $X∼\text{MVN}\left(\underset{∼}{\mu },\Omega =\sum^{-1}\right)$ with $X_{i}$ denoting the gene expression value of the i -th gene node. A zero-entry in the precision matrix Ω corresponds to conditional independence and no connecting line between nodes. In other words, if the off-diagonal (i,j) -th element $\omega_{ij}$ in Ω is zero, then the partial correlation ${|}_{ij}=cor\left(X_{i},X_{j}|X_{-\left(i,j\right)}\right)$ is zero; namely, the $X_{i}$ and $X_{j}$ are conditionally independent given the remaining variables, and there exists no edge between these paired nodes in the network. Therefore, under GGM, the problem of network construction becomes the inference of a sparse precision matrix or the selection of non-zero partial correlation.

Recent work on inferring network structure with GGM can be categorized into two groups. Methods in the first group focus on determining if an edge exists between nodes using the idea of “covariance selection”. When p is large, these methods follow the principle of variable selection with a regularization procedure to complete the binary decision about whether $\omega_{ij}$ or ${|}_{ij}$ is zero. Various methods of this regularization approach have been developed that adopt different objective functions and/or $L_{1}$ penalty, including neighborhood selection with lasso (M&B) by Meinshausen and Buhlmann (2006), graphical lasso (Glasso) in Friedman et al. (2008), the space partial correlation estimation (SPACE) in Peng et al. (2009), and the constrained $l_{1}$ minimization for inverse matrix estimation (CLIME) in Cai et al. (2011). These penalized optimization methods can be applied straightforwardly, but they are not designed to infer the intensity of edges or to interpret the dependence between nodes, although this information may be influential in biological experiments (Ni et al., 2020). If the inference, such as the estimation of the non-zero partial correlation, is based on a given network, then the network structure needs to be fixed first with one of the methods mentioned above. Therefore, this estimation procedure relies heavily on the choice of the selected network structure, which may cause concern about subsequent inference if the validity of this structure is in question.

Methods in the second group, usually under the Bayesian framework, explicitly adopt the uncertainty in the network graph, through a prior on the precision matrix, such as the G-Wishart, spike-and-slab lasso (SSL), and a subset-specific prior (Wang and Pillai, 2013; Mohammadi and Wit, 2015; Gan et al., 2019; Williams 2021; JalaliKhare and Michailidis, 2022). To enhance computational efficiency, researchers have proposed various tools, such as the double Metropolis-Hasting algorithm and birth-death Markov chain Monte Carlo methods, and the Bayes EM to estimate the maximum *a posteriori* (MAP) to avoid complex computation. These analyses provide a posterior probability for each candidate graph and a posterior inclusion probability for each edge. The inclusion probability, in this case, can be a good indication of its existence, but the strength of the edge is not considered in the computation. One solution may be to average the estimates of precision matrices in an element-wise way and weigh by the posterior probability of the matrix and the corresponding candidate graph. For instance, the BDgraph in Mohammadi and Wit (2015) can be utilized to perform this analysis. The computational burden in these procedures is fairly heavy due to the large number of nodes and the even more significant number of candidate graphs.

To relieve the computational burden, Gan et al. (2019) proposed a novel EM algorithm, called BAGUS, that first estimates the maximum *a posteriori* (MAP) of the precision matrix and then approximates the probability of edges with the precision matrix fixed at the MAP to learn the graph structure. BAGUS outperformed existing methods in terms of computation time, accuracy in recovering graph structure, and prediction error of the precision matrix. However, the uncertainty of the network graph and the posterior distribution of the edges are not accounted for in the BAGUS algorithm.

The inference of the strength of the edges has not been the target of these aforementioned algorithms. This inference requires a fully Bayesian approach and can be complicated in computation. In a recent research, Williams (2021) discussed the importance and implication of this topic. In that study, the edge inference was carried out with a fully Bayesian approach and the posterior probability of the precision element is used to infer the dependence between nodes. The conjugate Wishart prior was adopted to save computation time. If the SSL prior with a latent variable indicating the randomness in the edge existence is considered, further computational complexity will be incurred.

This research adopts the Bayesian learning approach for its ability to incorporate *a priori* information and to offer probabilistic inference, and for its wide application in bioinformatic research, including the Bayesian scoring rule for metabolite molecules (Ludwig et al., 2018), peak calling with Hi-C data (Xu et al., 2016), and pathway prioritization with posterior probability (Lin et al., 2018). The rationale of this research is twofold. First, an informative metric to quantify the strength of an edge is needed, which can provide more information beyond its existence. This is crucial when decoding the interplay between nodes or prioritizing intervention in a gene regulatory network. Second, since most genes do not work alone, the strength or intensity of the relationship between any two nodes should account for the presence of other genes when learning the network structure of a given set of genetic nodes. In this study, we start with the Bayesian MRF combining the conditional autoregressive (CAR) model to estimate the strength of the edge and its existence probability. Under the Gaussian CAR model, the conditional mean $E\left(X_{j}|X_{\left(-j\right)}\right)$ is expressed as $\underset{k\neq j}{\sum }\beta_{jk}X_{k}$ for j=1,2,...,p , where $X_{\left(-j\right)}≜\left\{X_{k}:k\neq j\right\}$ represents the set containing all variables except $X_{j}$ . Following Besag (1974) and Besag and Kooperberg (1995), the coefficient $\beta_{jk}$ is a function of elements in the precision matrix Ω , and is connected to the partial correlation ${|}_{jk}$ between $X_{j}$ and $X_{k}$ . That is, the $\beta_{jk}$ can be used to characterize the strength of dependence between these two genes. In addition, the Spike-and-Slab Lasso (SSL) prior proposed by Ročková and George (2018) is adopted for $\beta_{jk}$ . Then, the regularization procedure on these $\beta_{jk}$ ’s functions similarly to the “covariance selection” procedure in previous literature and provides a direct and intuitive interpretation of the intensity and relationship between nodes.

The rest of this article is organized as follows. The rationale and complete model of the Bayesian MRF and the implementation of prior knowledge are introduced in Section 2. In Section 3, extensive simulation studies are conducted to demonstrate the performance of the proposed model and comparison with other state-of-the-art methods. In Section 4, the proposed model is applied to a glioblastoma study with gene expression values from TCGA (Hutter and Zenklusen, 2018). Some biologically relevant findings will be highlighted. We then conclude with a discussion.

## 2 Methods

### 2.1 Learning network structure

To introduce the proposed Bayesian Markov Random field (BMRF) model, we first let the n×p matrix X represent the observed gene expression values of the p genes from the n subjects, where $x_{ij}$ is the expression value of the j -th gene ( j=1,2,…,p ) from the i -th subject ( i=1,2,…,n ). Without loss of generality, the values across subjects per gene are standardized so that $E\left(X_{j}\right)=0$ and $Var\left(X_{j}\right)=1$ . Under GGM, the p− dimensional random vector ${\left(X_{1},X_{2},\ldots ,X_{p}\right)}^{T}$ follows a multivariate normal distribution (MVN) with the following conditional distribution (Besag 1974),

$$X_{j}|X_{\left(-j\right)}∼N\left(\underset{k\neq j}{\sum }\beta_{jk}X_{k},\sigma_{j}^{2}\right),j=1,2,\ldots ,p.$$ (1)

Following Besag (1974) and Besag and Kooperberg (1995), the coefficients can be expressed as $\beta_{jk}=\frac{-\omega_{jk}}{\omega_{jj}}$ if j≠k . This is related to the partial correlation ${|}_{jk}$ between $X_{j}$ and $X_{k}$ where ${|}_{jk}=\frac{-\omega_{jk}}{\sqrt{\omega_{jj}\omega_{kk}}}$ . When the diagonal elements in Ω are equal, then $\beta_{jk}=\beta_{kj}$ and the underlying coefficients in the CAR model can be expressed as $\underset{∼}{\beta }=\left\{\beta_{jk}:1\leq j<k\leq p\right\}$ where $\left\|\underset{∼}{\beta }\right\|=p\left(p-1\right)/2$ is the number of unknown parameters to be estimated. Moreover, when $\beta_{jk}=0$ , the corresponding ${|}_{jk}=0$ , implying no edge between two gene nodes. These properties provide two advantages in supporting $\beta_{jk}$ as promising candidates in inferring the network structure. First, the selection of non-zero elements of $\beta_{jk}\in \underset{∼}{\beta }$ is equivalent to the decision of the existence of the edge. Second, the magnitude of these coefficients can quantify the relative intensity of the partial correlation between nodes. Their estimates can be derived based on the CAR model and thus the regression model. Such an approach would be easier than directly estimating the correlation coefficient matrix, especially when a direct estimate of the matrix is not straightforward due to the curse of dimensionality and the requirement of positive definiteness.

This CAR model is more general than those used in spatial statistics, where only neighboring “areas” are included in the mean structure. Here all genetic nodes are included first as a fully connected model. Then the procedures and computations below will decide which $\beta_{jk}$ remain and how strong the evidence is. In addition, this conditional distribution is also similar to node-wise regression where constraints are imposed to ensure symmetry in the $\beta_{jk}$ ’s (Ha et al., 2021).

### 2.2 Spike-and-slab lasso prior: Probabilistic estimation of edge

For the inference of $\beta_{jk}$ , we consider the Spike-and-Slab Lasso (SSL) prior (Rockova and George, 2018),

$$\pi \left(\beta_{jk}|\gamma_{jk}\right)=\gamma_{jk}\times \psi_{1}\left(\beta_{jk}\right)+\left(1-\gamma_{jk}\right)\times \psi_{0}\left(\beta_{jk}\right).$$ (2)

where the slab distribution $\psi_{1}\left(\beta_{jk}\right)=\frac{\tau_{1}}{2}\exp\left(-\tau_{1}\left|\beta_{jk}\right|\right)$ and the spike $\psi_{0}\left(\beta_{jk}\right)=\frac{\tau_{0}}{2}\exp\left(-\tau_{0}\left|\beta_{jk}\right|\right)$ are both double exponential (Laplace) with a small $\tau_{1}$ and large $\tau_{0}$ , respectively. The binary $\gamma_{jk}$ takes the value of one if $\beta_{jk}$ represents a large effect, and $\gamma_{jk}=0$ if the effect is around zero. Therefore, the marginal posterior probability of $\gamma_{jk}=1$ can represent the probability of the edge existence.

The SSL prior is considered a fundamental variable selection tool in the Bayesian framework for sparse models. This differs from the previously mentioned penalized optimization methods for variable selection, where the estimated effect size is biased. In addition, the SSL prior is flexible because it allows the shrinkage effects to vary among different edges. For instance, a substantial shrinkage penalty can be deployed for those edges with weak partial correlation, while for those with strong partial correlation, a non-shrinkage effect can be considered. Other studies have used the SSL prior in the matrix inference (Peterson et al., 2015; Deshpande et al., 2019; Gan et al., 2019). For instance, Gan et al. (2019) assumed this prior for the off-diagonal entries in the precision matrix, the $\omega_{jk}$ in our case, and Deshpande et al. (2019) adopted this prior for the regression parameter, the $\beta_{jk}$ in our case. In Peterson et al. (2019), the SSL prior was incorporated to model the network similarity.

By adopting the SSL prior, we can select the influential edges and perform statistical inference with $\beta_{jk}$ . The BMRF model specification is completed with a Bernoulli prior for $\gamma_{jk}$ , $\gamma_{jk}∼Ber\left(p_{jk}\right)$ , where $p_{jk}$ follows a conjugate beta distribution. Specifically, in contrast to previous studies investigating if the edge exists, here we are interested in constructing the posterior distributions of $\beta_{jk}$ and $\gamma_{jk}$ , respectively, to model the strength of the edge and its existence probability.

### 2.3 Computation

Since the posterior distributions of $\gamma_{jk}$ and $\beta_{jk}$ are the bases of the probabilistic inference, one can obtain the posterior samples of $\gamma_{jk}$ and $\beta_{jk}$ with Markov chain Monte Carlo (MCMC) methods implemented in any standard Bayesian software. In the following simulation studies and applications, the R package *R2OpenBUGS* is used to carry out the computations.

When the number of gene nodes is large, the number of possible edges and parameters increases rapidly. Fortunately, most genetic networks/pathways are sparse. For instance, the sparsity of the signaling pathway networks in KEGG ranges between 5% and 10%. Liu et al. (2009), Zhao et al. (2012), and Mohammadi and Wit (2015) have adopted similar values in their simulation studies. Such *a priori* information can be utilized in a p×p adjacency matrix $G^{*}$ , where elements $g_{jk}=1$ if two genes $X_{j}$ and $X_{k}$ are known biologically to be associated and $g_{jk}=0$ otherwise. By imposing the matrix of domain knowledge $G^{*}$ on $\underset{∼}{\beta }=\left\{\beta_{jk}:1\leq j<k\leq p\right\}$ , one can save computational cost from estimating the edges known to be non-existent. Similarly, another p×p adjacency matrix $M^{*}$ can be introduced to contain elements $m_{jk}=1$ if the corresponding interrelation is of interest to particular experts. This would force the inclusion of the edge in the network, yet the flexibility remains when later inference does not favor its existence. Inclusion of these two matrices and the distribution of $p_{jk}$ can account for all the cases described here. For example, this matrix $M^{*}$ can be derived first and the data-driven prior on $\gamma_{jk}$ can be further established. The BMRF with this setup will be denoted as BMRF.P in later sections.

## 3 Numerical simulation experiments

For performance evaluation and comparison with existing methods, three types of network graph are considered in the simulation studies: the random network (M1), random scale-free network (M2), and fixed network structure (M3). In M1, edges are considered exchangeable, and all nodes in a network are equally important. The scale-free network in M2 is commonly adopted for genetic pathways, where the edges are not exchangeable because hub nodes may exist in the network. These two are designed to compare with the traditional approach of variable selection, where only the number of true edges successfully detected is of concern. While in M3, with a fixed and known structure, further comparison between the inclusion probability in previous Bayesian methods and the existence probability in current BMRF can be carried out, and the strength of edge is demonstrated. In other words, in M3, in addition to the number of true edges successfully detected, both the probability of existence and strength of edges will be emphasized.

### 3.1 Simulation settings

In the random network setting M1, the GGM is generated with the following steps, similar to the procedures in Fan et al. (2009), and Peng et al. (2009).

1) Set up the network sparsity S, 0≤S≤1

2) Construct the true network E by randomly sampling the Bernoulli $e_{ij}$ with probability S . If $e_{ij}=1$ , then there is an edge between the node i and j , and 0 otherwise.
3) Generate the precision matrix $\Omega =\left(\omega_{ij}\right)$ according to E by

$$\omega_{ij}=\left\{ \begin{array}{l} 1,\;i=j\\ 0,\;i\neq j,\;e_{ij}=0\\ U\left(W\right),\;i\neq j,\;e_{ij}=1 \end{array}\right.$$

where W=[−1,−0.05]∪[0.05,1] and U(.) denotes the uniform distribution.

4) To assure the positive definiteness of Ω , each off-diagonal $\omega_{ij}$ in Ω is replaced by the original $\omega_{ij}$ divided by $1.5\times \overset{p}{\underset{j=1,j\neq i}{\sum }}\left|\omega_{ij}\right|$ .
5) Average the rescaled matrix calculated in (4) with its transpose matrix to ensure symmetry. The values of the nodes are generated from a multivariate normal distribution (MVN) with a zero mean vector and the precision matrix.

Note that different combinations of p and S have been considered, denoted as M1.1 for p=25, S=0.05 , M1.2 for p=25, S=0.10 , M1.3 for p=50, S=0.05 , and M1.4 for p=50, S=0.10 . The number of edges in each network is about $\left(\begin{array}{l} p\\ 2 \end{array}\right)\times S$ .

In the random scale-free network setting M2, the R package huge was used to generate the scale-free networks. Two settings (M2.1) p=25 and (M2.2) p=50 were considered. The average number of edges in the scale-free network is p−1 .

In M3, the fixed network structure setting, a scale-free network graph containing 50 nodes and 49 edges was selected, and the node values were generated with the *huge* package with the partial correlation in the network set at −0.216.

For all stimulations, the hyper-parameters were specified as $\tau_{1}=2$ and $\tau_{0}=20$ , the sample size was n=250 , and the number of replications in each setting was 100. More detailed information, including the network sparsity and number of true edges, is summarized in the Supplementary Table S1. For the proposed BMRF, the corresponding edge is selected for the network if the posterior probability of $\gamma_{jk}=1$ is greater than 0.5. This choice is used in simulation studies when comparing different regularized methods for variable selection.

### 3.2 Comparing methods and evaluation criteria

The proposed BMRF model was compared with M&B, Glasso, SPACE, and CLIME, as well as with the Bayesian approach BDgraph using the Bayesian model averaging procedure (denoted as BD_BMA), the Maximum a posterior probability procedure (BD_MAP), and BAGUS. M&B and Glasso were performed with the R package huge, and the tuning parameter used in these two methods was chosen through the rotation information criterion (ric). The SPACE approach was performed with the R package space with the tuning parameter set by default. The package flare was used for the estimator CLIME with tuning parameters obtained by 5-fold cross-validation. The R package BDgraph was used for BDgraph. BAGUS was performed with the R code provided in the online supplementary material in Gan et al. (2019).

Several criteria were used to compare performance, including the total number of true positives (TP), the sensitivity (SEN), the specificity (SPE), the false discovery rate (FDR), the Matthew correlation coefficient (MCC), and the F1-score (F1). These quantities are calculated based on TP and the total number of false negatives (FN), where TP is defined as the total number of true edges that were successfully identified, and FN as the total number of true edges that failed to be detected.

### 3.3 Implementations

When handling a large set of gene nodes with BMRF, we recommend two modeling strategies, one with a non-informative prior and the other with a data-driven prior. The former is denoted as BMRF.O, corresponding to the prior distribution $\gamma_{jk}∼Ber\left(p_{jk}\right)$ with $p_{jk}$ from a beta distribution with mean 0.5. The latter, denoted as BMRF.P, models the network edges with $p_{jk}∼Beta\left(\alpha^{*},\beta^{*}\right)$ , an informative prior with a mean larger than 0.5 if $e_{ij}\in M^{*}\cap G^{*}$ , or $p_{jk}∼Beta\left(\alpha^{†},\beta^{†}\right)$ , a non-informative prior with a mean around 0.5. As stated earlier, the matrices $M^{*}$ and $G^{*}$ can be elicited by experts, with domain knowledge, with a screening scheme based on sparsity or sample correlation, or with SPACE proposed in Peng et al. (2009), which outperforms other methods when dealing with a scale-free network structure. In the following analysis, the matrix $G^{*}$ containing the edges corresponding to the largest 10% absolute sample correlations was determined first when the network sparsity was set at 0.05 (or the top 15% if set at 0.10). For the matrix $M^{*}$ , we incorporated the information from SPACE to accelerate the computational efficiency. The mean of the informative prior $p_{jk}∼Beta\left(\alpha^{*},\beta^{*}\right)$ was set at 0.8.

### 3.4 Results

#### 3.4.1 Existence or not: Random network (M1 and M2)

To compare performance, Table 1 lists the values of several evaluation criteria under settings M1.1, M1.3, and M2.2. A quick look shows that, except for BD_MAP, the other four Bayesian algorithms perform equivalently or slightly better than the rest. In most cases, BAGUS is the best in terms of F1-score and MCC, but is less satisfactory in the number of true positives (TP) and sensitivity (SEN). Other Bayesian algorithms achieve larger TP and sensitivity. Among the Bayesian methods, BD_BMA and BD_MAP tended to identify more edges, leading to larger TP and SEN but lower F1 and MCC. Consequently, these two often produce a larger FDR. BD_MAP was usually the worst in this regard due to the lack of consideration of model uncertainty. In M1.3 and M2.2, BAGUS, M&B and SPACE perform similarly well. Generally, the proposed BMRF.O and BMRF.P are comparable to the best performers. The performances under other settings are displayed in the Supplementary Table S2.

**TABLE 1:** Values of six evaluation criteria (F1, MCC, FDR, TP, SEN, and SPE) under simulation settings M1.1, M1.3, and M2.2. Each value is the average of 100 replications with standard error (SE) in parentheses.

M1.1	F1	MCC	FDR	TP	SEN	SPE
BMRF.O	0.89 (0.06)	0.89 (0.07)	0.12 (0.09)	13.6 (3.0)	0.91 (0.08)	0.99 (0.005)
BMRF.P	0.88 (0.06)	0.87 (0.06)	0.16 (0.09)	13.8 (3.1)	0.92 (0.07)	0.99 (0.005)
BD_BMA	0.87 (0.06)	0.86 (0.06)	0.19 (0.09)	14.1 (3.1)	0.94 (0.07)	0.99 (0.006)
BD_MAP	0.58 (0.09)	0.60 (0.08)	0.58 (0.10)	14.1 (3.2)	0.94 (0.07)	0.93 (0.020)
BAGUS	0.94 (0.05)	0.94 (0.05)	0.02 (0.04)	13.6 (2.9)	0.91 (0.08)	0.99 (0.002)
Glasso	0.83 (0.06)	0.82 (0.06)	0.25 (0.09)	14.1 (3.2)	0.94 (0.07)	0.98 (0.009)
CLIME	0.88 (0.08)	0.89 (0.08)	0.01 (0.02)	11.9 (2.5)	0.81 (0.13)	0.99 (0.001)
M&B	0.90 (0.06)	0.90 (0.06)	0.10 (0.09)	13.8 (3.0)	0.92 (0.07)	0.99 (0.005)
SPACE	0.89 (0.06)	0.88 (0.06)	0.14 (0.08)	13.8 (3.0)	0.92 (0.07)	0.99 (0.005)

**M1.3**	**F1**	**MCC**	**FDR**	**TP**	**SEN**	**SPE**
BMRF.O	0.78 (0.05)	0.78 (0.05)	0.13 (0.05)	44.2 (3.7)	0.72 (0.07)	0.99 (0.002)
BMRF.P	0.79 (0.05)	0.79 (0.05)	0.14 (0.04)	45.6 (4.0)	0.74 (0.07)	0.99 (0.002)
BD_BMA	0.76 (0.04)	0.75 (0.05)	0.25 (0.06)	47.7 (4.2)	0.77 (0.06)	0.99 (0.004)
BD_MAP	0.50 (0.03)	0.51 (0.04)	0.63 (0.03)	48.8 (4.3)	0.79 (0.06)	0.93 (0.007)
BAGUS	0.80 (0.05)	0.80 (0.05)	0.04 (0.03)	42.3 (3.8)	0.69 (0.07)	0.99 (0.001)
Glasso	0.78 (0.05)	0.78 (0.05)	0.10 (0.05)	43.0 (3.7)	0.70 (0.08)	0.99 (0.002)
CLIME	0.63 (0.11)	0.67 (0.09)	0.01 (0.02)	29.2 (6.7)	0.48 (0.11)	0.99 (0.001)
M&B	0.79 (0.05)	0.80 (0.05)	0.04 (0.03)	41.9 (3.4)	0.68 (0.08)	0.99 (0.001)
SPACE	0.80 (0.05)	0.80 (0.05)	0.09 (0.04)	43.9 (3.8)	0.71 (0.07)	0.99 (0.002)

**M2.2**	**F1**	**MCC**	**FDR**	**TP**	**SEN**	**SPE**
BMRF.O	0.78 (0.09)	0.77 (0.09)	0.24 (0.06)	39.2 (6.1)	0.80 (0.13)	0.99 (0.002)
BMRF.P	0.84 (0.04)	0.84 (0.04)	0.23 (0.04)	45.3 (2.8)	0.92 (0.06)	0.99 (0.002)
BD_BMA	0.83 (0.04)	0.83 (0.04)	0.27 (0.05)	46.0 (2.3)	0.94 (0.05)	0.99 (0.003)
BD_MAP	0.52 (0.03)	0.55 (0.03)	0.64 (0.02)	45.7 (2.3)	0.93 (0.05)	0.93 (0.006)
BAGUS	0.89 (0.05)	0.89 (0.05)	0.04 (0.03)	41.5 (3.6)	0.85 (0.07)	0.99 (0.01)
Glasso	0.83 (0.06)	0.82 (0.07)	0.19 (0.07)	41.7 (4.1)	0.85 (0.08)	0.99 (0.004)
CLIME	0.63 (0.11)	0.65 (0.09)	0.52 (0.13)	47.3 (2.1)	0.97 (0.04)	0.95 (0.025)
M&B	0.88 (0.05)	0.88 (0.05)	0.08 (0.05)	41.6 (4.2)	0.85 (0.09)	0.99 (0.002)
SPACE	0.86 (0.05)	0.86 (0.05)	0.16 (0.05)	43.7 (3.1)	0.89 (0.06)	0.99 (0.003)

**FIGURE 1:**
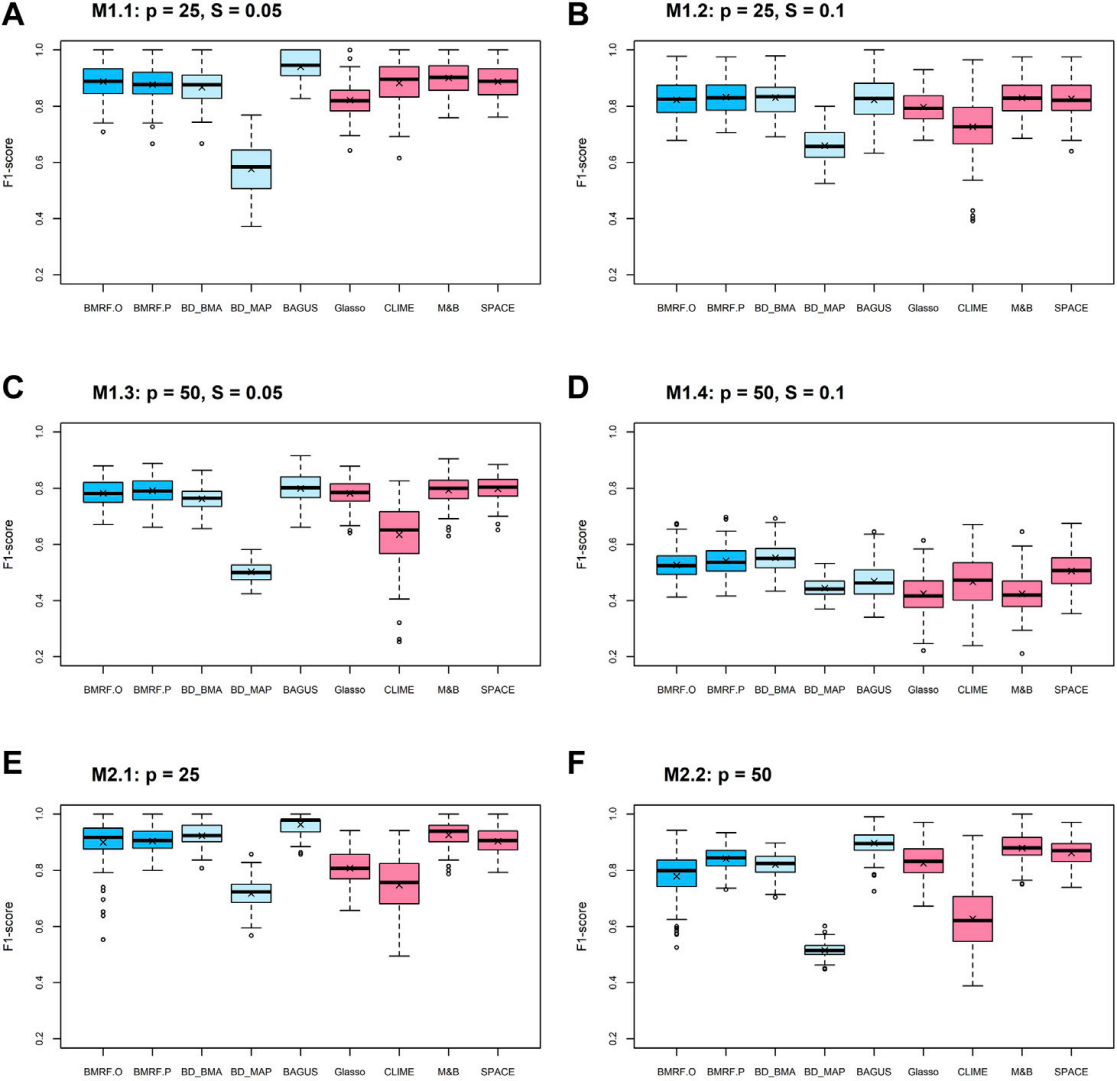
Boxplots of F1-scores from 100 replications under each method. Each subfigure corresponds to a setting with a combination of p and S . Note that the blue boxplots correspond to the five Bayesian algorithms and the pink ones correspond to the four penalized methods.

One metric among the evaluation criteria, the F1-score, is displayed in Figure 1. When the number of nodes p is as large as 50, most methods are still satisfactory if the graph is sparse, such as when the case sparsity = 0.05 in Figures 1C,F. The Bayesian approaches, both BMRF and BDgraph, tend to identify more edges when compared with the frequentist approach to variable selection, therefore leading to a higher F1-score. These results highlight the advantages of probabilistic inference on the conditional dependence in network analysis, in contrast to the detection of whether or not the edge exists.

#### 3.4.2 Existence probability: Fixed network (M3)

**FIGURE 2:**
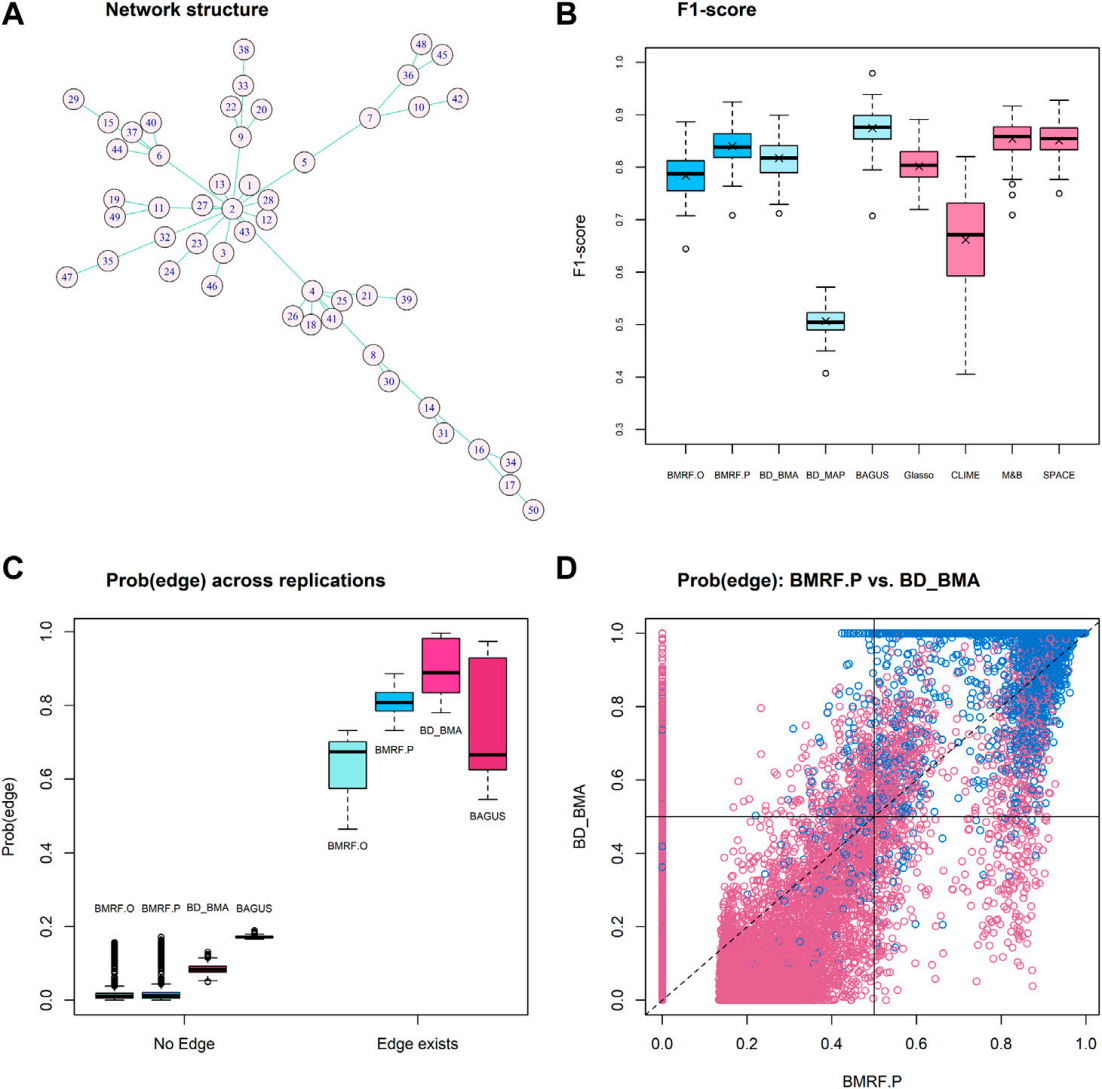
Results of competing methods under simulation setting M3. **(A)** The true network structure with 49 true edges; **(B)** Boxplots of the F1-score from 100 replications under each method; **(C)** Boxplots of average existence probability over 100 replications under each of the four Bayesian algorithms. The left group No Edge corresponds to the case when there is truly no edge, and the right group Edge exists corresponds to the case when the edge truly exists. Each boxplot in the right group is composed of 49 average probabilities; **(D)** The edge existence probability from BMRF.P *versus* the inclusion probability from BD_BMA for each edge across replications. Blue circles indicate true edges and red indicates no edge. The vertical and horizontal solid lines denote the cut-off values for BMRF.P and BD_BMA, respectively.

In setting M3, a fixed network structure with two hub nodes was determined first, as shown in Figure 2A, and then the node values were generated from MVN. The numbers of edges connecting to the two hubs, Node-2 and Node-4, are 14 and 7, respectively. Various methods were then applied to infer the network structure. Across 100 replications, the average number of edges estimated by each method is listed in Table 2. Four methods, BMRF.P, BD_BMA, M&B, and SPACE, performed the best, with the first two being slightly better with a smaller standard error. When examining the F1-score in Figure 2B, BAGUS performed best.

**TABLE 2:** The listed values are the average number of estimated edges connecting to each of the two hub nodes (Node-2 and Node-4) across 100 replications under M3. The number in parenthesis is the standard error. The true number of edges connecting to Node-2 is 14 and to Node-4 is 7.

	Node-2 (true = 14)	Node-4 (true = 7)
BMRF.O	8.5 (1.3)	5.7 (1.0)
BMRF.P	14.1 (0.8)	7.0 (0.9)
BD_BMA	14.4 (0.8)	7.2 (0.8)
BD_MAP	16.4 (1.5)	9.5 (1.6)
BAGUS	14.1 (0.4)	6.3 (0.7)
Glasso	14.7 (0.8)	7.6 (1.8)
CLIME	17.6 (2.3)	9.9 (1.9)
M&B	14.3 (0.6)	6.5 (1.2)
SPACE	14.7 (0.9)	6.8 (1.1)

For the probabilistic inference of edge existence, we first stratify the edges into two groups, truly *No Edge* and *Edge exists*, and display in Figure 2C the estimated edge existence probability or the inclusion probability derived from the four competing methods, BMRF.O, BMRF.P, BD_BMA, and BAGUS. As indicated in the figure, when there exists no edge (labeled *No Edge* on *X*-axis in the figure), BMRF.O and BMRF.P provide very low probabilities while BD_BMA and BAGUS show slightly larger probabilities. When the edge truly exists, labeled *Edge exists* on *X*-axis in the right group in the figure, the BD_BMA performs the best and is followed by BMRF.P. It needs to be clarified, however, that it may not be fair to compare the edge existence probability against the inclusion probability because of the different definitions. In BMRF, the existence probability of the edge is the posterior probability of $\gamma_{jk}=1$ ; while in BD_BMA, the inclusion probability is the sum of all posterior probabilities of networks containing the edge. The inclusion probability in this sense can be viewed as the expected value of the existence probability if all possible network structures are accounted for. In BAGUS, the inclusion probability is estimated with a conditional probability, conditioning on the Bayes EM estimates of the other parameter values. In other words, the BAGUS estimate assumes a fixed network structure rather than estimating across all possible structures.

The association between the existence probability from BMRF.P and the inclusion probability from BM_BMA is further examined in Figure 2D. The blue circles represent true edges and the red circles indicate non-existent edges. These two are fairly consistent, except that BD_BMA seems to detect more non-existent edges (red circles) than BMRF.P. The values of the other criteria are summarized in the Supplementary Table S3.

#### 3.4.3 Accuracy of probabilistic inference

An alternative way to evaluate the probabilistic inference of the edge existence is the Brier score (Brier, 1950), which can be calculated for each of the Bayesian estimates. The Brier score, ranging between 0 and 1, is a mean squared difference between the true class label (edge exists or not) and the estimated probability. Smaller values of the Brier score indicate better estimates. This score has become a common measure to assess the accuracy of the probabilistic estimates of binary outcomes, especially when comparing performance of machine learning algorithms (Dinga et al., 2019; Ovadia et al., 2019).

**FIGURE 3:**
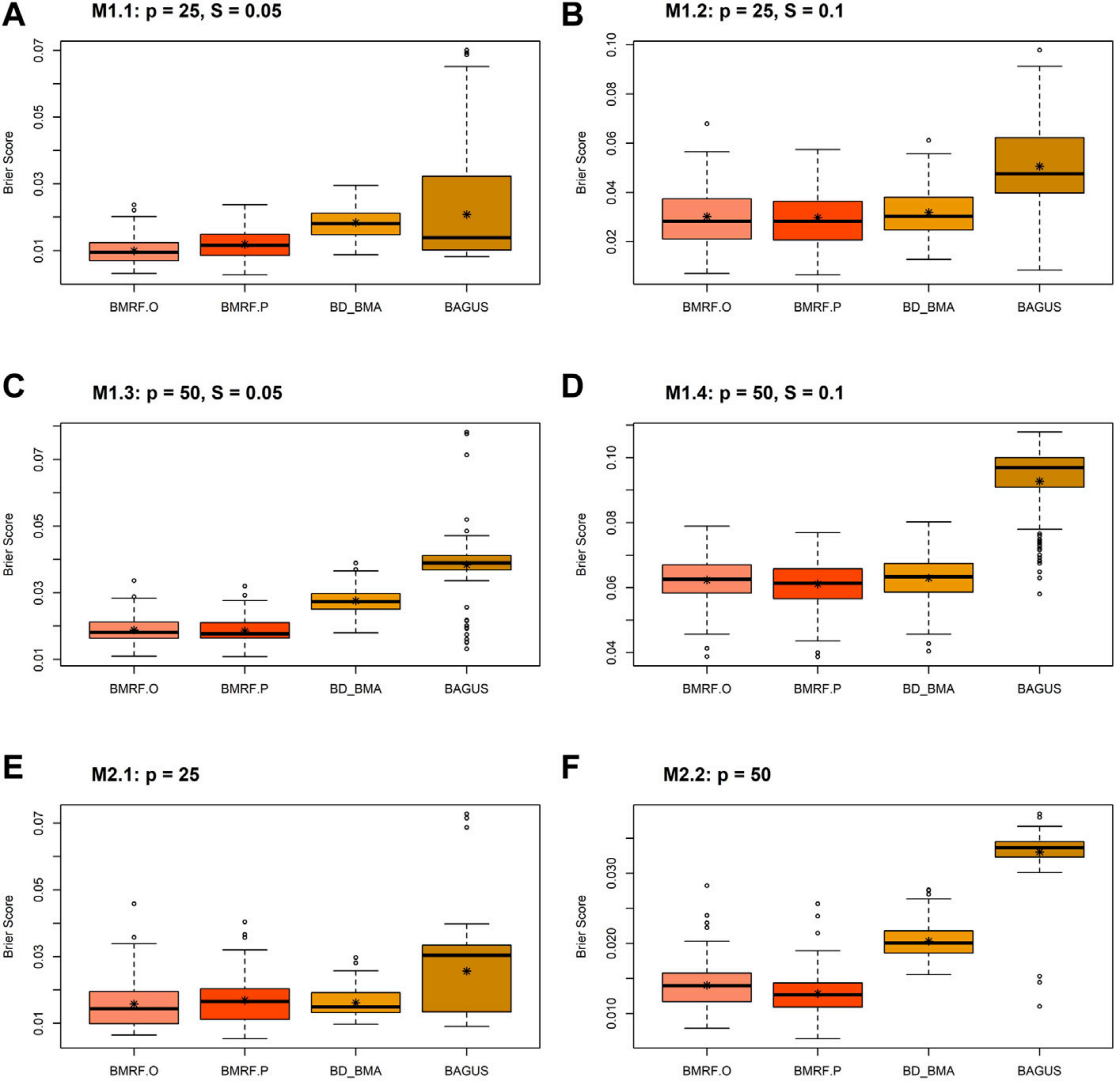
Boxplots of the Brier scores of the four Bayesian estimates, BMRF.O, BMRF.P, BD_BMA, and BAGUS, under six different simulation settings: **(A)** M1.1; **(B)** M1.2; **(C)** M1.3; **(D)** M1.4; **(E)** M2.1; **(F)** M2.2.

The boxplots of the Brier score for the four Bayesian estimates under different simulation settings are displayed in Figure 3. Every boxplot is composed of 100 Brier scores, each from a replication in the simulations. In all the subfigures, it can be observed that all four methods provide small Brier scores, mostly below 0.07, indicating good accuracy. In other words, they provide large probability estimates when the edge truly exists and small probability estimates when the edge does not exist. This pattern is consistent with that in Figure 2C under simulation setting M3. Note that the average Brier scores of the four Bayesian estimates under M3 are 0.01, 0.01, 0.02, and 0.03 for BMRF.O, BMRF.P, BD_BMA, and BAGUS, respectively. The second observation in the figure is that the probabilistic estimates of BAGUS are more variable and usually slightly larger than the rest. This could result from the utilization of MAP in the BAGUS probability estimate, where the estimate is a probability conditioning on MAP estimates of the other parameters and therefore incurs further estimation errors in the graph structure.

## 4 Applications in two glioblastoma studies

In this section, we consider two data types, array and sequencing gene expression values, collected from Glioblastoma (GBM) patients. GBM is a grade IV malignant brain tumor, usually in adults. After being diagnosed, patients have a median survival time of about 12–15 months and generally respond poorly to treatments (Stupp et al., 2005; The Cancer Genome Atlas Research Network, 2008). Although several molecular biomarkers have been identified, such as TP53 mutation and overexpression in EGFR (Bralten and French, 2011; Zhang et al., 2018), targeted therapy shows a limited effect (Shergalis et al., 2018; Banerjee et al., 2021). Recent interest has focused on the molecular mechanism of the Janus kinase/signal transducer and activator of transcription (JAK-STAT) signaling pathway (Jain et al., 2012; Ou et al., 2021).

Here we aim at constructing relationships within two networks, EGFR and JAK-STAT, based on RNA sequencing and array data, respectively. The BMRF model is applied to two pathways to examine the conditional dependence among gene nodes and detect influential molecular relationships to understand the underlying biological mechanism better. The expression values were downloaded from the University of California Santa Cruz (UCSC Xena) TCGA Hub and TCGA GDC data portal. The array gene expressions were generated from the Affymetrix HT Human Genome U133a microarray platform with mRNA values in the log two scale, and the sequencing data from Illumina HTSeq. The nodes in the JAK-STAT network were collected with the procedures in Chang et al. (2020). The EGFR network was determined based on the protein-protein interaction (PPI) network in STRING. The final array data consist of 27 gene expression values from 253 primary tumor tissues, and the sequencing data contain 30 genes from 83 tissues. All are primary tumor tissues from male patients aged 40 and 75. The procedures (computing sample correlation, SPACE, and taking union) discussed earlier were carried out and resulted in 99 possible edges in the JAK-STAT network and 80 edges in the EGFR network, respectively, as the starting sets of edges for further analysis. More information about the selection procedures is in the Supplementary Sections S2, S3.

### 4.1 Edges in the JAK-STAT network with gene expression arrays

**FIGURE 4:**
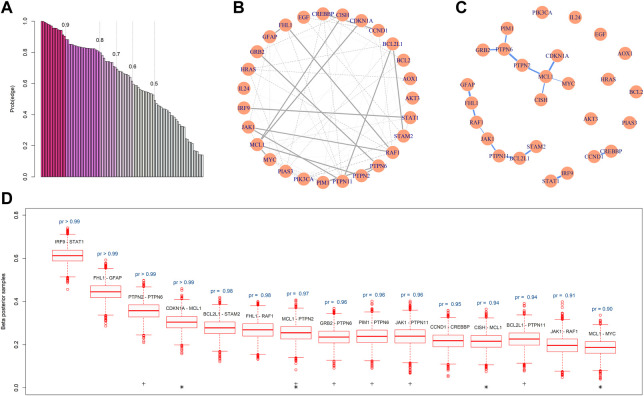
Gene regulatory network constructed by BMRF.P. **(A)** The ordered probabilities of the 99 edges are estimated by BMRF.P, and different colors correspond to different thresholds. The first 69 are the edges with a probability greater than 0.5; **(B)** The estimated genetic network. The 15 thick lines are edges with an estimated existence probability greater than 0.9; **(C)** The network structure containing only the 15 edges, where the width of the edge corresponds to the magnitude of the existence probability; **(D)** Boxplots of the posterior samples of the strength coefficient corresponding to each one of the 15 edges. The text above the boxplot represents the estimated existence probability. The ‘+’ indicates edges involving genes in the PTPN family and ‘*’ involves *MCL1*.

Based on the GBM array data, the BMRF.P identified 69 edges in the network with probabilities greater then 0.5, 15 of which were associated with a posterior existence probability greater than 0.9. Figure 4A plots the posterior probabilities of all 99 edges, from the largest to the smallest. Figure 4B shows the resulting gene regulatory network, where the 15 edges are represented with thick lines and the others with thin lines. The corresponding magnitudes of the 15 existence probabilities are displayed in Figure 4C, where the width denotes the magnitude of the probability. The boxplots in Figure 4D show the posterior samples of the strength of each edge, all displaying positive conditional correlations between paired nodes. This is consistent with the pattern of co-expression, and the first two pairs seem to be strongly correlated with each other.

Note that the ordered existence probabilities in Figure 4A may be useful if prioritization is of interest. When comparing the top leading 15 edges with the lines in KEGG, we note that two edges (*JAK1*-*PTPN11* and *IRF9*-*STAT1*) are listed in KEGG. These two each have a probability greater than 0.95. The other thirteen edges with such a large probability were not listed in KEGG and may deserve further validation and investigation. For the connecting lines in KEGG, the BMRF posterior probabilities can be adopted to provide relative degrees of conditional dependence.

The proposed BMRF detected several influential biomarkers and biomarker pairs in the JAK-STAT network. First, the node *MCL1* is clearly crucial in this network since it appears in four edges (indicated with ‘*’) among the 15 in Figure 4C. This hub node has been reported as one of the cell apoptosis inhibitors associated with the progression of GMB and participates in the signaling of the maintenance of neural stem cells (Fassl et al., 2012; Murphy et al., 2014). Second, in the constructed network by BMRF.P, the *PTPN2*, *PTPN6*, and *PTPN11* in the Protein-Tyrosine Phosphatase Non-Receptor (PTPN) family play critical roles. They appear in six edges (indicated with ‘+’) among the 15 in Figure 4C. This is not surprising since the expression level of the immunotherapy target PTP2 has been shown to associate with the grade of glioma (Wang et al., 2018). Liu and others (Liu et al., 2011) have suggested *PTPN11* as a functional target for treating glioblastomas in human and animal studies, and Cerami et al. (2010) have identified *PTPN11* as associated with an oncogenic process in GBM patients. Members of the PTPN family induce dephosphorylation of *JAK*, thereby regulating JAK-STAT signaling (Xu and Qu, 2008; Jain et al., 2012; Hammarén et al., 2019). Third, the top-ranking pair shows the largest conditional dependence between *IRF9* and *STAT1*. This interaction was found to involve in type I interferon (IFN) signaling and anti-viral immune response (Au-Yeung et al., 2013). Fourth, BMRF.P identified the relationship between *MYC* and *MCL1*, where the transcription factor c-Myc of *MYC* was associated with the regulation the proliferation and survival of glioblastoma stem cells (Wang et al., 2008; Ha et al., 2015).

Other summary statistics regarding these 15 edges and all 69 edges are provided in the Supplementary Table S4; Supplementary Figure S3, respectively; and other interactions are summarized in the Supplementary Table S5. The findings of BMRF.P are compared with those of alternative procedures in the Supplementary Figures S4, S5. All edges identified by BMRF. P overlap with those identified by other procedures. Similar to the simulation studies, the edges identified by CLIME and BD_BMA overlap the least with the other procedures. This demonstrates again that the BMRF.P can provide more information than previous algorithms.

### 4.2 Edges in EGFR network with RNA-Seq

**FIGURE 5:**
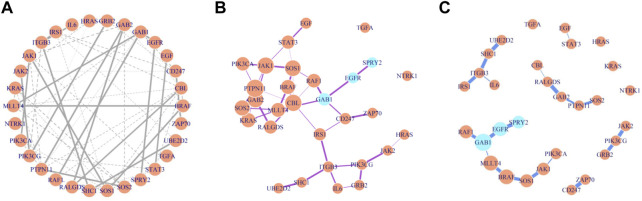
Gene regulatory network constructed by BMRF.P based on different thresholds. The width of edges is proportional to the existence probability and node size in **(B)** and **(C)** is proportional to number of immediate neighbors. **(A)** These 55 edges are of estimated existence probability greater than 0.5; **(B)** These 41 edges are of estimated existence probability greater than 0.7; **(C)** The 20 edges are of estimated existence probability greater than 0.9.

The BMRF model was next applied to the RNA sequencing gene expression of the 30 genes in the EGFR network. Figures 5A–C demonstrate the structure and relative strength of edges among these gene nodes, when different thresholds for the probability of existence are adopted. For instance, with the 0.5 threshold, 55 edges were identified, and with 0.90, 20 edges were detected. Three genes, *GAB1*, *EGFR*, and *SPRY2*, are colored differently to indicate that relatively *EGFR* depends more on the other two, if the conditional dependence inside this network is quantified and prioritized. Studies have shown that *GAB1* is involved in the cell proliferation and signaling process of positive feedback activation to *EGFR* (Kapoor and DM O’Rourke, 2010; Azuaje et al., 2015) and *SPRY2* knockdown is related to the negative prognosis and drug resistance of GBM (Walsh et al., 2015; Park et al., 2018; Day et al., 2020).

Another interesting observation is about the genes *GAB1* and *GAB2*. These two are crucial in the constructed network, appearing in five edges among 20 (Supplementary Figure S7). The probability of connection between these genes is strong (>0.9). The *GAB1* is connected to *EGFR* in the lower left in Figure 5C, and *GAB2* appears in the middle in Figure 5C. They apparently deserve more attention when studying the activity of this network.

In addition, note in Figure 5B where both *PTPN11* and *CBL* have six neighbors and are displayed with larger circles, indicating more connection with other gene nodes. When examining the edges with an existence probability greater than 0.9 in Figure 5C, these two genes interact with *GAB2*, *RALGDS*, and *SOS2* (in the middle of Figure 5C). These genes have been reported in the literature to associate with immune function and GBM. The findings here are not just reproducible results but also support that further investigation in the collective effect of these genes may be warranted. The hub nodes identified here and by other methods are consistent, as listed in Supplementary Table S7. More details can be found in Supplementary Section S3.

## 5 Discussion

In addition to the binary decision of edge existence, the proposed BMRF algorithm offers a probability measure of this existence, and is able to quantify the relative strength of edges, through the conditional autoregressive model and SSL prior. Its novelty lies in the Bayesian inference of the relative strength of the edges so that the conditional dependence can be prioritized. Simulation studies have demonstrated that, for the scale-free network, the performance of BMRF can be significantly improved when prior information is incorporated. Even when only the existence is of interest, the BMRF model can provide performance comparable with existing methods. In the two glioblastoma studies, the proposed algorithm highlights highly dependent subsets in the network that are worth for further investigation.

In contrast to other Bayesian network approaches, BMRF focuses on inference of the relative strength of the conditional dependence, while others are more interested in identifying non-zero elements in the precision matrix (Huang, 2022). The proposed method provides a complimentary tool when more interpretations of the relationship among genes is needed. That is, this BMRF can be executed with other Bayesian models, including ones that assign for the precision matrix a prior distribution composed of a product of all probability distributions of each element (Wang, 2012; Peterson et al., 2013; Gan et al., 2019), so that the post-processing computation can be saved. Another good choice is the BAGUS algorithm proposed by Gan et al. (2019). It provides a fast and accurate estimate of the graph structure, including the MAP estimate of the precision matrix with EM and the approximate inclusion probability of each edge. The implementation of the frequentist perspective may increase the scalability of BMRF. For example, these estimates may be utilized as baseline information to determine which edges to initially include for the inference of edge strength, or to tune the hyper-parameter values in the prior distributions of $\beta_{jk}$ and $\gamma_{jk}$ . Incorporation of such information may reduce the number of iterations required in the MCMC algorithm to save computational burden. The choice of the hyperparameter values $\tau_{0}$ and $\tau_{1}$ in the prior distribution does not change the basic outcome. The posterior distributions of $\beta_{jk}$ corresponding to different hyperparameters are very similar, leading to the same conclusions based on the posterior distributions. Similarly, the order of the relative strength remains the same. In other words, the prioritization is not affected by the hyperparameter values. The magnitudes of the existence probability are linearly correlated, though the value may differ slightly. These observations are based on our limited experiments with the GBM application. Further studies may be warranted.

The computation time for the BMRF can be as long as 30 min per replication, especially under the current R package *R2OpenBUGS*. This is slow and can hinder the use of the proposed model. In contrast, the computation for the frequentist methods discussed here and the BAGUS is much faster. This is a reason why we did not consider a graph with more than 100 nodes in simulation studies. This limitation also restricts the use of the BMRF model to screen pairwise relationship among a large group of genes. Further research in tailoring a fast computation algorithm is worth investigating.

The proposed algorithm can be extended to integrative network analysis. With a graphical model comprised of biomarkers from different platforms, it is possible to reveal the underlying complex biological structure among various forms of molecules (Peng et al., 2010; Yin and Li, 2011; Ha et al., 2021). In this case, adjustments in the CAR model would be needed to account for the genetic variables at different levels. However, this approach would be computationally intensive when facing the enormous number of all parameters combined.

Another generalization of the BMRF is to relax the distributional assumption in the CAR model. The GGM for the gene network assumes the MVN as the joint distribution, and the conditional and marginal distribution are also Gaussian. This assumption may not be valid generally, particularly for gene expression data. Ho et al. (2022) performed a systematic study to investigate the multivariate normality of gene expression values. Several parametric and nonparametric multivariate tests were considered and applied on more than twenty sets of empirical data. It was concluded that the normality assumption is not guaranteed. Classical research has addressed non-Gaussian Markov random fields (Besag, 1974), but these studies are not designed for sparse neighborhood selection. One solution would be to combine the non-paranormal distribution in Liu et al. (2009) or the exponential family graphical model (Yang et al., 2015) with BMRF for further investigation.

When comparing the relative strength estimated by BMRF with the connecting lines in current pathway/network databases, two issues should be noted. First, databases like KEGG collect current knowledge of relationships, such as interactions and reactions, between molecules, and the resulting pathways/networks represent a collection of research findings from multiple studies involving various types of genetic markers. These studies are not necessarily comparable. In other words, although KEGG can be a good source to examine if the conditional dependence detected by BMRF has been identified before, one should bear in mind that the comparison may not be fair, since the data sets as well as the genetic biomarkers can be very different. Second, since the curation of pathways/networks is based on published literature, the definition of their connecting lines differs from the existence probability and the inclusion probability considered in this study. Therefore, a validation study of the findings here, especially for the two GBM studies, would need to be carefully designed. Disease status, tissue sample source and conditions, and genetic markers would all need to be incorporated for consideration.

## Data Availability

Publicly available datasets were analyzed in this study. This data can be found here: The glioblastoma dataset can be downloaded from TCGA hub and GDC hub in https://xenabrowser.net/datapages/. The R code for the implementation is available in https://github.com/YJGene0806/BMRF_Code.
