# Supplementary material for: Probabilistic edge inference of gene networks with markov random field-based bayesian learning
Source: Front Genet. 2022 Nov 10;13:1034946. doi: 10.3389/fgene.2022.1034946 (PMC9684306; doi:10.3389/fgene.2022.1034946)
Supplement: Supplementary file 1 [file DataSheet1.pdf]

Supplementary materials for “Probabilistic Edge Inference of Gene Networks with Markov Random Field-based Bayesian Learning”

Supplementary section S1: Supporting information for simulation studies

Table S1. Details of simulation settings. For example, in setting (M1.1), the average number of true edges across 100 replications is around 15.

Setting		# of true edges	Sparsity
(M1.1) Random network	P = 25	≈15	0.05
(M1.2) Random network	P = 25	≈30	0.10
(M1.3) Random network	P = 50	≈62	0.05
(M1.4) Random network	P = 50	≈123	0.10
(M2.1) Scale-free network	P = 25	24	0.08
(M2.2) Scale-free network	P = 50	49	0.04
(M.3) Fixed network	P = 50	49	0.04

Table S2. Values of evaluation criteria under simulation settings M1.2, M1.4, and M2.1. Each value is the average of 100 replications with standard error (SE) in parentheses. The false discovery rate (FDR) is defined as the average value of FDP.

M1.2	F1	MCC	FDR	TP	SEN	SPE
Glasso	0.80 (0.06)	0.78 (0.06)	0.18 (0.08)	23.2 (3.1)	0.78 (0.10)	0.98 (0.010)
CLIME	0.73 (0.11)	0.74 (0.10)	0.01 (0.03)	17.4 (4.1)	0.59 (0.10)	0.99 (0.004)
M&B	0.83 (0.06)	0.82 (0.07)	0.09 (0.05)	22.8 (3.1)	0.77 (0.10)	0.99 (0.005)
SPACE	0.83 (0.06)	0.81 (0.07)	0.10 (0.05)	23.0 (3.2)	0.77 (0.10)	0.99 (0.006)
BMRF.O	0.82 (0.07)	0.81 (0.07)	0.07 (0.05)	22.1 (3.0)	0.74 (0.10)	0.99 (0.005)
BMRF.P	0.83 (0.06)	0.82 (0.06)	0.11 (0.05)	23.4 (3.1)	0.78 (0.09)	0.99 (0.006)
BD_BMA	0.83 (0.06)	0.82 (0.06)	0.12 (0.06)	23.7 (3.1)	0.80 (0.09)	0.99 (0.007)
BD_MAP	0.65 (0.06)	0.62 (0.07)	0.45 (0.07)	23.8 (3.1)	0.80 (0.09)	0.93 (0.020)
BAGUS	0.82 (0.08)	0.82 (0.07)	0.02 (0.03)	21.2 (2.7)	0.72 (0.11)	0.99 (0.002)
M1.4	F1	MCC	FDR	TP	SEN	SPE
Glasso	0.43 (0.07)	0.49 (0.06)	0.06 (0.05)	34.5 (6.1)	0.28 (0.07)	0.99 (0.002)
CLIME	0.47 (0.09)	0.50 (0.06)	0.12 (0.10)	41.0 (12.2)	0.33 (0.10)	0.99 (0.009)
M&B	0.43 (0.07)	0.49 (0.05)	0.06 (0.04)	34.2 (5.4)	0.28 (0.06)	0.99 (0.002)
SPACE	0.51 (0.07)	0.53 (0.05)	0.12 (0.05)	44.1 (5.1)	0.36 (0.06)	0.99 (0.003)
BMRF.O	0.53 (0.05)	0.54 (0.05)	0.17 (0.05)	48.0 (4.0)	0.39 (0.05)	0.99 (0.003)
BMRF.P	0.54 (0.05)	0.55 (0.05)	0.19 (0.05)	50.3 (4.6)	0.41 (0.06)	0.99 (0.003)
BD_BMA	0.56 (0.05)	0.55 (0.05)	0.23 (0.05)	54.0 (4.0)	0.44 (0.05)	0.99 (0.004)
BD_MAP	0.45 (0.03)	0.38 (0.04)	0.57 (0.03)	58.2 (4.3)	0.47 (0.05)	0.93 (0.006)
BAGUS	0.47 (0.06)	0.52 (0.05)	0.07 (0.04)	38.9 (4.5)	0.32 (0.05)	0.99 (0.002)
M2.1	F1	MCC	FDR	TP	SEN	SPE
Glasso	0.81 (0.06)	0.80 (0.06)	0.31 (0.09)	23.5 (0.7)	0.98 (0.03)	0.96 (0.017)
CLIME	0.75 (0.09)	0.75 (0.09)	0.39 (0.12)	23.8 (0.5)	0.99 (0.02)	0.94 (0.033)
M&B	0.93 (0.04)	0.92 (0.05)	0.12 (0.07)	23.6 (0.7)	0.98 (0.03)	0.99 (0.008)
SPACE	0.90 (0.04)	0.90 (0.04)	0.16 (0.06)	23.6 (0.7)	0.98 (0.03)	0.98 (0.008)
BMRF.O	0.90 (0.08)	0.89 (0.08)	0.11 (0.07)	21.8 (2.2)	0.91 (0.09)	0.99 (0.007)
BMRF.P	0.90 (0.04)	0.90 (0.04)	0.16 (0.07)	23.6 (0.6)	0.98 (0.03)	0.98 (0.008)
BD_BMA	0.92 (0.04)	0.92 (0.04)	0.13 (0.06)	23.7 (0.5)	0.99 (0.02)	0.99 (0.007)
BD_MAP	0.72 (0.06)	0.72 (0.06)	0.43 (0.07)	23.5 (0.7)	0.98 (0.03)	0.93 (0.019)
BAGUS	0.96 (0.03)	0.96 (0.04)	0.02 (0.03)	22.7 (1.4)	0.95 (0.06)	0.99 (0.003)

Table S3. Values of evaluation criteria under simulation setting M3. Each value is the average of 100 replications with standard error (SE) in parentheses. The false discovery rate (FDR) is defined as the average value of FDP.

M3	F1	MCC	FDR	TP	SEN	SPE
Glasso	0.80 (0.04)	0.80 (0.04)	0.19 (0.07)	39.3 (2.9)	0.80 (0.06)	0.99 (0.004)
CLIME	0.66 (0.09)	0.68 (0.08)	0.48 (0.11)	46.9 (2.0)	0.96 (0.04)	0.96 (0.020)
M&B	0.85 (0.04)	0.85 (0.04)	0.08 (0.05)	39.2 (2.9)	0.80 (0.06)	0.99 (0.002)
SPACE	0.85 (0.04)	0.85 (0.04)	0.16 (0.05)	42.3 (2.6)	0.86 (0.05)	0.99 (0.002)
BMRF.O	0.78 (0.04)	0.78 (0.04)	0.22 (0.05)	38.7 (2.5)	0.79 (0.05)	0.99 (0.003)
BMRF.P	0.84 (0.04)	0.84 (0.04)	0.22 (0.05)	44.5 (2.2)	0.91 (0.05)	0.99 (0.003)
BD_BMA	0.82 (0.04)	0.82 (0.04)	0.27 (0.04)	45.2 (2.1)	0.92 (0.04)	0.99 (0.003)
BD_MAP	0.51 (0.03)	0.54 (0.03)	0.65 (0.02)	44.6 (2.0)	0.93 (0.04)	0.93 (0.005)
BAGUS	0.88 (0.04)	0.87 (0.04)	0.04 (0.03)	39.5 (2.8)	0.81 (0.06)	0.99 (0.001)

Supplementary section S2: Supporting information for array-based gene expression data from the TCGA glioblastoma study.

The set of edges corresponding to the top 15% sample correlation and edges identified by SPACE were first derived and then the union of the two sets was taken, leading to a set of 99 edges and a sparsity around 0.3.

Figure S1. Sample correlation matrix for the array-based gene expression data.

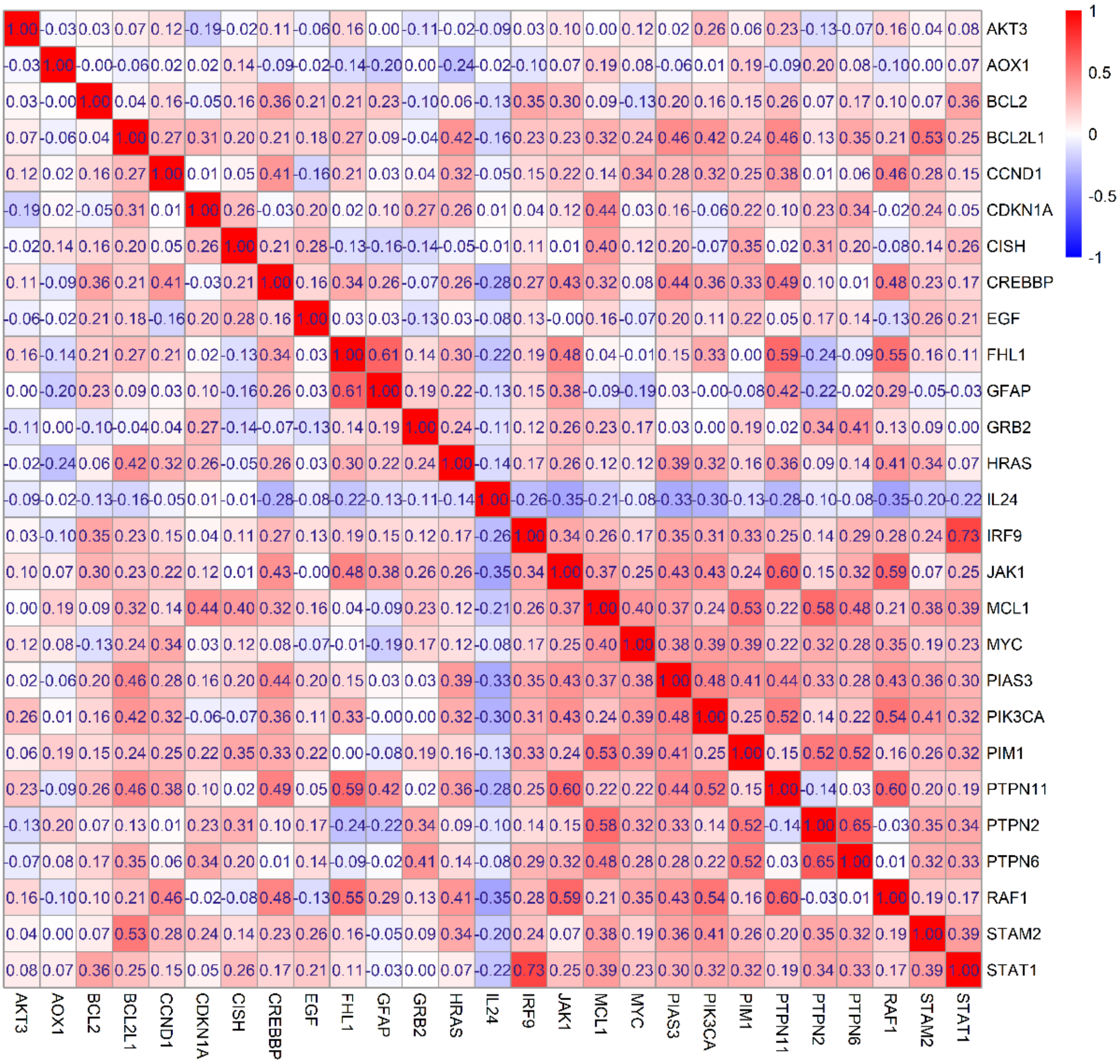

Figure S2. Venn diagram of the set of edges passing the screening with sample correlation and the set of edges identified by SPACE in the GBM array study. SPACE identified 91 edges and sample correlation screened the top 53 edges ($\binom{27}{2} = 351; 351 \times 0.15 \approx 51$). The intersection contains 45 edges in common.

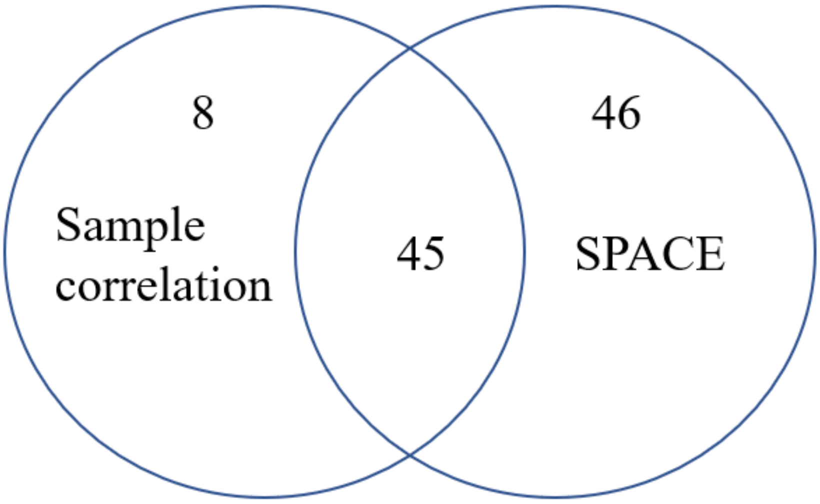

Figure S3. The gene regulatory network constructed by BMRF.P. The width of each edge represents the magnitude of the posterior mean of $\underset{\sim}{\beta}$. Lines colored in red (blue) represents positive (negative) partial correlation.

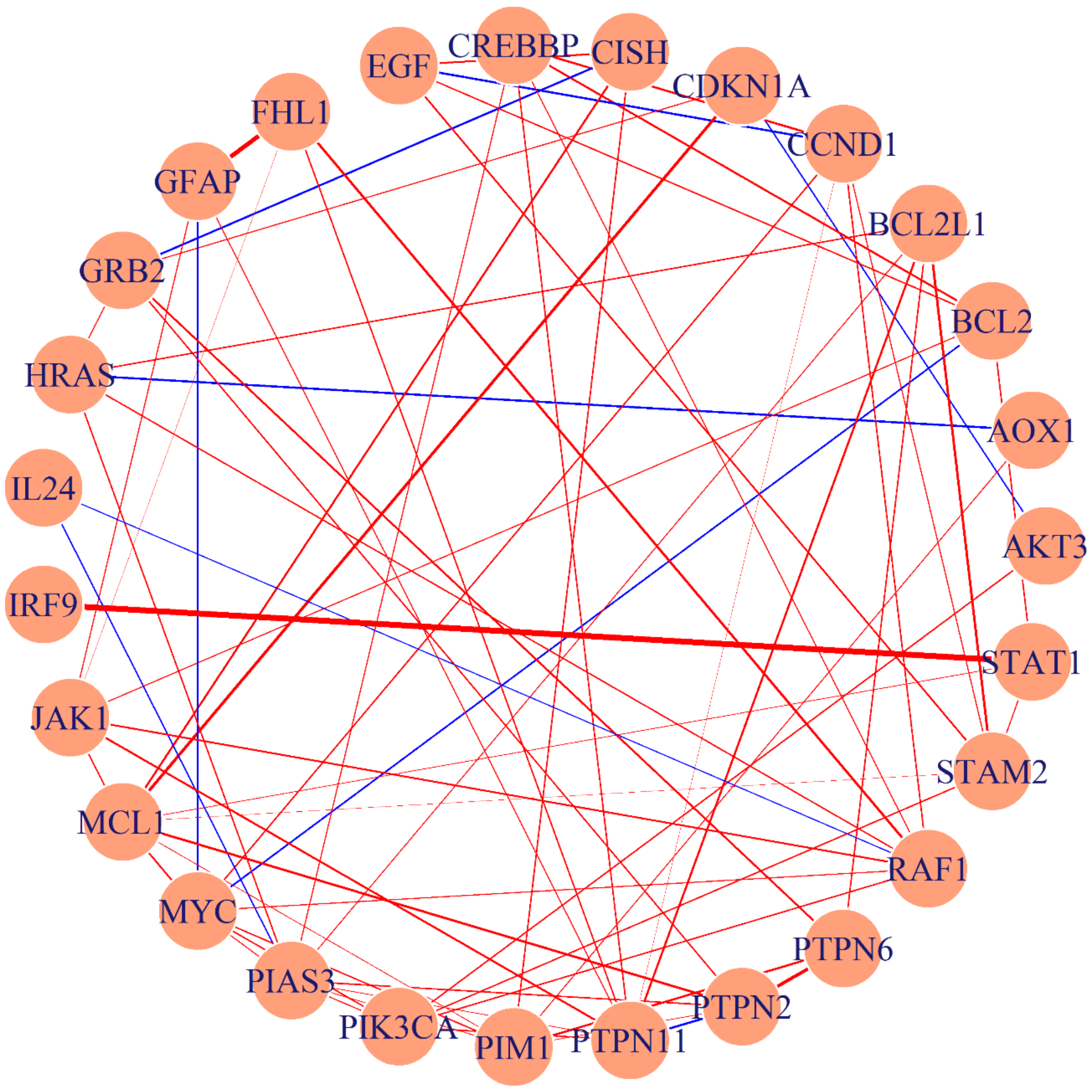

Table S4. Details about the 15 edges (existence probability > 0.9).

(1) Gene1, Gene2: the two genes connected by the selected edge

(2) Prob(edge): the estimated probability of existence

(3) Beta.mean: the posterior mean

(4) Beta.SD: the standard deviation of the beta posterior samples

(5) Q2.5%, Q97.5%, and C.I. width: the quantiles and width of the 95% credible interval of beta

Gene1	Gene2	Prob(edge)	Beta.Mean	Beta.SD	Q2.5%	Q97.5%	C.I. width
IRF9	*STAT1*	>0.99	0.61	0.04	0.54	0.69	0.15
FHL1	*GFAP*	>0.99	0.45	0.04	0.37	0.52	0.15
PTPN2	*PTPN6*	>0.99	0.36	0.04	0.28	0.44	0.16
CDKN1A	*MCL1*	0.99	0.30	0.04	0.23	0.38	0.15
BCL2L1	*STAM2*	0.98	0.28	0.04	0.20	0.36	0.16
FHL1	*RAF1*	0.98	0.27	0.04	0.19	0.35	0.17
MCL1	*PTPN2*	0.97	0.26	0.04	0.17	0.34	0.17
GRB2	*PTPN6*	0.96	0.24	0.04	0.16	0.31	0.16
PIM1	*PTPN6*	0.96	0.24	0.04	0.16	0.32	0.17
JAK1	*PTPN11*	0.96	0.24	0.05	0.15	0.33	0.18
CCND1	*CREBBP*	0.95	0.22	0.04	0.14	0.30	0.15
CISH	*MCL1*	0.943	0.22	0.04	0.14	0.29	0.16
BCL2L1	*PTPN11*	0.942	0.23	0.04	0.15	0.31	0.16
JAK1	*RAF1*	0.913	0.20	0.05	0.11	0.29	0.18
MCL1	*MYC*	0.905	0.19	0.04	0.11	0.26	0.15

Figure S4. Venn diagram of sets containing edges identified by BMRF.P, M&B, BD_BMA, and SPACE.

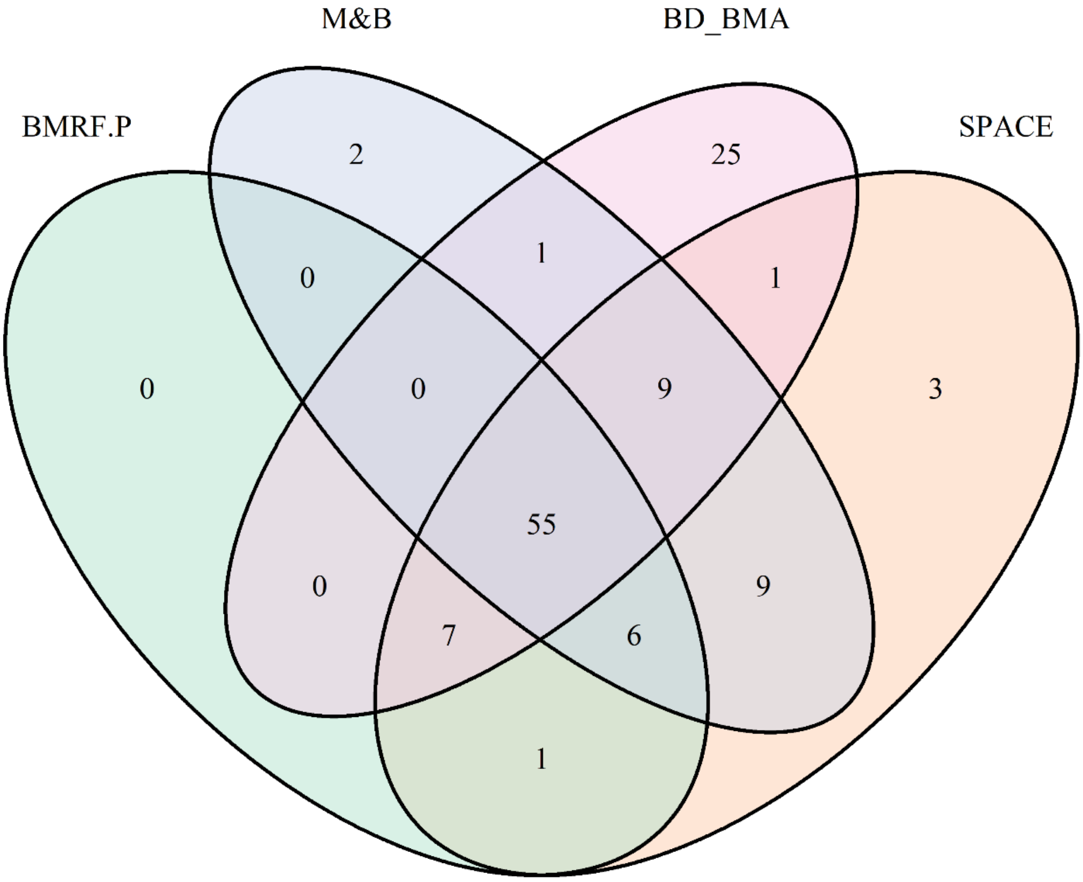

Figure S5. Numbers of identified edges.

Numbers in the diagonal represent the number of edges identified by each method listed in the row or in the column.

Numbers in the upper triangle represent the number of common edges identified by both the column and row methods.

Numbers in the lower triangle represent the number of edges identified by the column method but not the row method.

For example, BMRF.P identifies 69 edges. Among them, 61 edges are identified by M&B as well, while 8 among the 69 are identified by BMRF.P but not by M&B.

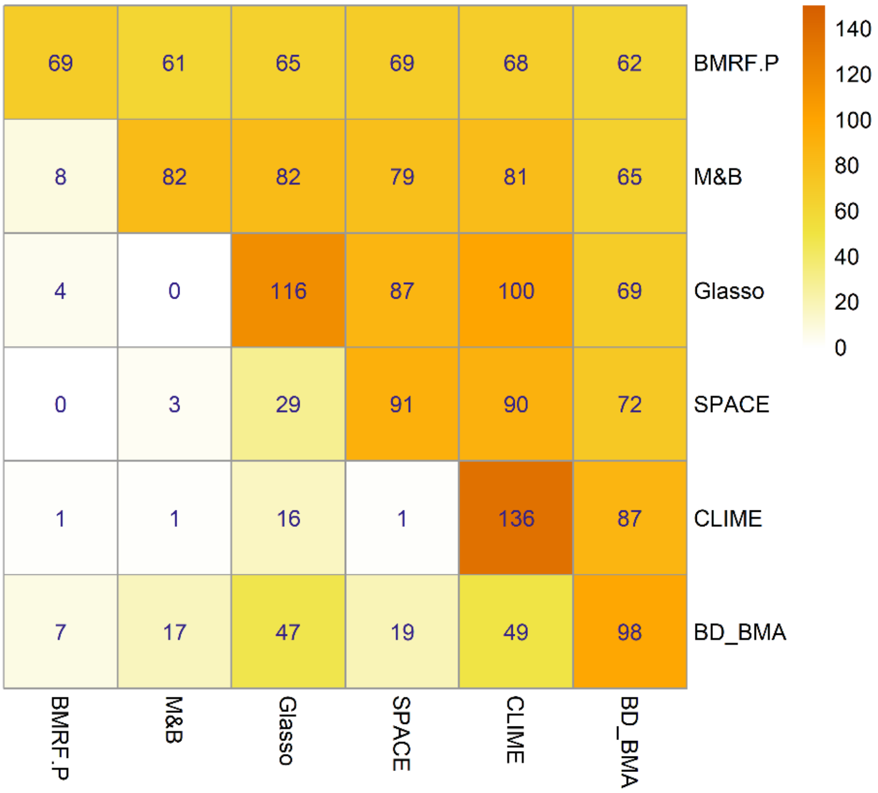

Table S5. List of selective interactions that were reported before. The symbol "✓" indicates an edge that was successfully identified by the corresponding column method, and the symbol "×" indicates a failure to be identified.

Gene1	Gene2	BMRF.P (prob. of existence)	BMRF.P (95% C.I. of beta)	M&B	Glasso	SPACE	CLIME	BD_BMA	KEGG	String	BioGrid
IRF9	*STAT1*	>0.99 (✓)	(0.54, 0.69)	✓	✓	✓	✓	>0.99 (✓)	✓	✓	✓
JAK1	*PTPN11*	0.96 (✓)	(0.15, 0.33)	✓	✓	✓	✓	0.99 (✓)	✓	✓	✓
RAF1	*HRAS*	0.83 (✓)	(0.07, 0.23)	✓	✓	✓	✓	>0.99 (✓)	✓	✓	✓
MYC	*BLC2*	0.80 (✓)	(-0.28, -0.13)	×	×	✓	✓	0.92 (✓)	×	×	✓

Supplementary section S3: Application of BMRF to analyze RNA sequencing data from TCGA glioblastoma dataset.

Here, the RNA sequencing data from glioblastoma (GBM) patients were used to construct gene regulatory networks. The expression data were first downloaded from TCGA GDC Data Portal, where the gene expression value with log2 scale of Fragments Per Kilobase per Million (FPKM) plus 1 was generated on Illumina HTSeq platform. Following the same procedures in previous data analyses, only primary tumor tissues from male patients between 40 ~ 75 years were included in the analysis. In this application, we extracted the protein-protein interaction (PPI) network from the STRING database (https://string-db.org/) version 11.0b (https://version-11-0b.string-db.org/) to select a gene set showing interaction with *EGFR*. *EGFR*, is commonly known as a frequent alteration and oncogenic molecule associated with GBM, and it has been the target clinical biomarker and prognostic factor in research (Heimberger et al., 2005; Eskilsson et al., 2018; Westphal et al., 2017). The aim is to estimate the regulatory relationship between genes in the PPI network around *EGFR* to examine the underlying biological mechanisms of GBM. The data for analysis consist of 30 genes and 83 primary tumor tissues.

All the implementations of BMRF.P are the same as before. The screening of possible interactions was first carried out by the data-driven procedures, where the union of the set containing lines of the top 15% sample correlation (the corresponding value is 0.41) and the set of lines identified by SPACE (57 edges) was derived. This results in a total of 80 edges and will be estimated by BMRF.P.

Figure S6 shows the regulatory network constructed by BMRF.P. In this case, BMRF.P identified 55 edges with $prob(edge) > 0.5$, and the corresponding network structure is shown in Figure S6 (a). In particular, there are 41 interactions computed with edge probability higher than 0.7, and 20 edges with existing probability greater than 0.9. The corresponding network structures constructed by the different probabilistic thresholds are presented in Figure S6 (b) and (c). By the strength of estimated existence probability, BMRF can prioritize the importance of identified edges so that we can highlight those highly evidential results in this analysis and construct the sub-network structure. Another merit of using probability to measure uncertainty is that the strength of probability is more interpretable and easy to implement. On the other hand, however, the magnitude of the tuning parameter of those lasso-based approaches, which can control the network sparsity, usually does not have a general criterion to interpret the exact value. The posterior distributions for the relative strength of those 20 edges with existence probability greater than 0.9 are illustrated by the violin plot in Figure S7. The posterior mean and 95% credible interval, which illustrate the relative partial correlation

of these 20 edges, are summarized in Table S6.

Table S7 and S8 compare the results analyzed by BMRF.P with other competing methods. Table S7 lists the top 3 hub nodes identified by each method. When comparing the results by adopting 0.5 as the selecting threshold, the hub nodes founded by BMRF.P are almost the same as other methods. However, when leveraging a stricter threshold, which means we have more confidence in the findings, BMRF.P highlights different hub nodes. In other words, the hub nodes pointed out by the competing methods as well as by BMRF.P with a 0.5 threshold tells us that these nodes connect more edges than others, but the intensity of these edges can be weak. Instead, using a rigorous threshold, such as the probability greater than 0.9, the hub nodes identified by BMRF.P can be interpreted as not only connecting more edges than others but also the corresponding partial correlation intensity is vital. This finding again highlights the usefulness of determine target genes via probabilistic prioritization.

Table S8 compares the similarity of findings between all methods. Consistent with the conclusion in simulation studies and the first application study, the findings from BMRF.P are usually more similar to M&B and SPACE, which showed better estimation performance against others in simulation studies. The Glasso and BM_BMA identified more and different edges than other methods, however, we found that these two methods tend to identify more false-positive signals in simulation studies.

We focus on those edges connected with the *EGFR* to gain some biologically meaningful findings. We first look at *GAB1*, which is also identified by BMRF.P as the hub node when adopting strict threshold. Much evidence shows that the *GAB1* is involved in the signaling process of positive feedback activation to *EGFR* and further engages in the response of enhancing cell proliferation (Kapoor and DM O'Rourke, 2010; Azuaje et al., 2015). Another interaction with *EGFR*, the *SPRY2*, which has been shown to associate with a negative prognostic indicator for the survival of GBM patients, several papers have reported that the effect of *SPRY2* knockdown on cell proliferation may drive the resistance to the *EGFR* inhibition and further associated with negative drug response (Walsh et al., 2015; Park et al., 2018; Day et al., 2020; TCGA 2008). These interactions can be served as potential targets for further research when focusing on *EGFR*-driven association to therapeutic targets of GBM. The regulatory network constructed by BMRF.P also identifies some important biomarkers. For example, the interaction between *GAB1* and *CD247*, which the higher expression level of *GAB1* may associate with shorter survival time for GMB patients (Liu et al., 2014) and the critical role of *CD247* in T cell activation highly related to the overall survival for cancer patients (Wang et al., 2019) has been demonstrated, can also be a new biological insight for further GBM research.

REFERENCES

Azuaje, F., Tiemann, K., and Niclou, S. P. (2015). Therapeutic control and resistance of the EGFR-driven signaling network in glioblastoma. *Cell Commun. Signal.* 13, 23. doi:10.1186/s12964-015-0098-6

Day, E. K., Sosale, N. G., Xiao, A., Zhong, Q., Purow, B., and Lazzara, M. J. (2020). Glioblastoma cell resistance to EGFR and MET inhibition can be overcome via blockade of FGFR-SPRY2 bypass signaling. *Cell Rep.* 30, 3383-3396.e7. doi:10.1016/j.celrep.2020.02.014

Eskilsson, E., Røsland, G. V., Solecki, G., Wang, Q., Harter, P. N., Graziani, G., et al. (2018). EGFR heterogeneity and implications for therapeutic intervention in glioblastoma. *Neuro-Oncol.* 20, 743–752. doi:10.1093/neuonc/nox191

Heimberger, A. B., Suki, D., Yang, D., Shi, W., and Aldape, K. (2005). The natural history of EGFR and EGFRvIII in glioblastoma patients. *J. Transl. Med.* 3, 38. doi:10.1186/1479-5876-3-38

Kapoor, G. S., and O'Rourke, D. M. (2010). SIRPα1 receptors interfere with the EGFRvIII signalosome to inhibit glioblastoma cell transformation and migration. *Oncogene* 29, 4130–4144. doi:10.1038/onc.2010.164

Liu, H., Li, G., Zeng, W., Zhang, P., Fan, F., Tu, Y., et al. (2014). Combined detection of Gab1 and Gab2 expression predicts clinical outcome of patients with glioma. *Med. Oncol.* 31, 77. doi:10.1007/s12032-014-0077-6

Park, J.-W., Wollmann, G., Urbiola, C., Fogli, B., Florio, T., Geley, S., et al. (2018) Sprouty2 enhances the tumorigenic potential of glioblastoma cells. *Neuro-Oncol.* 20, 1044–1054. doi:10.1093/neuonc/noy028

The Cancer Genome Atlas Research Network, (2008). Comprehensive genomic characterization defines human glioblastoma genes and core pathways. *Nature* 455, 1061–1068. doi:10.1038/nature07385

Walsh, A. M., Kapoor, G. S., Buonato, J. M., Mathew, L. K., Bi, Y., Davuluri, R.V., et al. (2015). Sprouty2 drives drug resistance and proliferation in glioblastoma. *Mol. Cancer Res.* 13, 1227–1237. doi:10.1158/1541-7786.MCR-14-0183-T.

Wang, Q., Li, P., and Wu, W. (2019). A systematic analysis of immune genes and Overall survival in cancer patients. *BMC Cancer* 19, 1225. doi:10.1186/s12885019-6414-6

Westphal, M., Maire, C. L., and Lamszus, K. (2017). EGFR as a target for glioblastoma treatment: an unfulfilled promise. *CNS Drugs* 31, 723–735. doi:10.1007/s40263-017-0456-6

Figure S6. Gene regulatory network constructed by BMRF.P based on different thresholds. The width of edges is proportional to the existence probability. (a) These 55 edges are of estimated existence probability greater than 0.5; (b) These 41 edges are of estimated existence probability greater than 0.7; (c) The 20 edges are of estimated existence probability greater than 0.9.

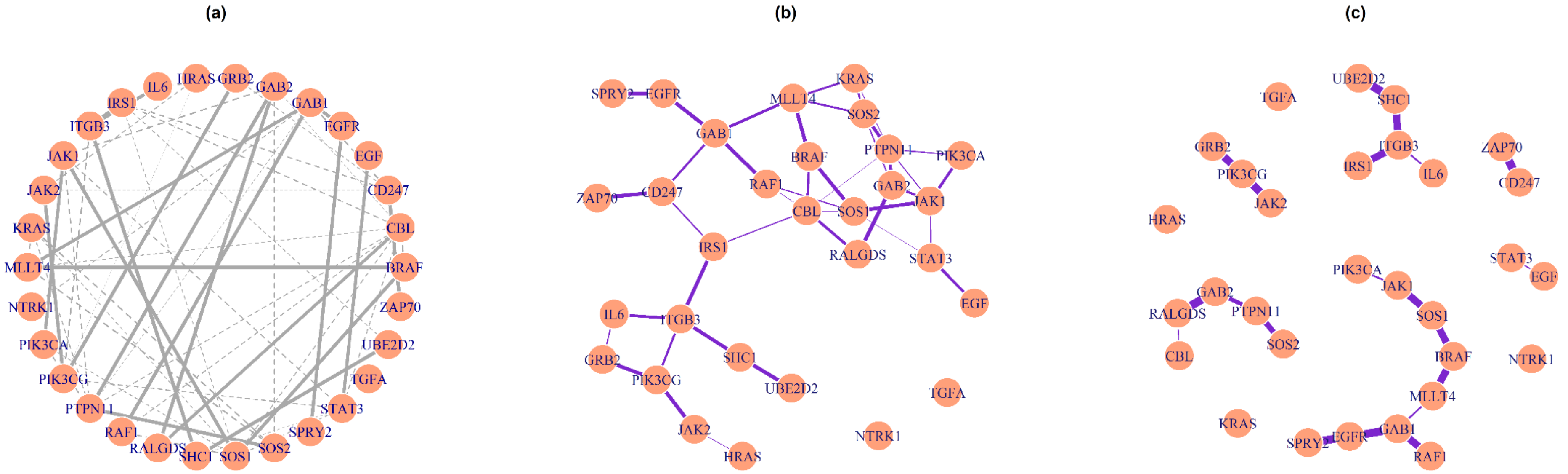

Figure S7. The violin plot represents the posterior distribution of relative intensity (β_{jk}) for the top 20 edges with existence probability greater than 0.9.

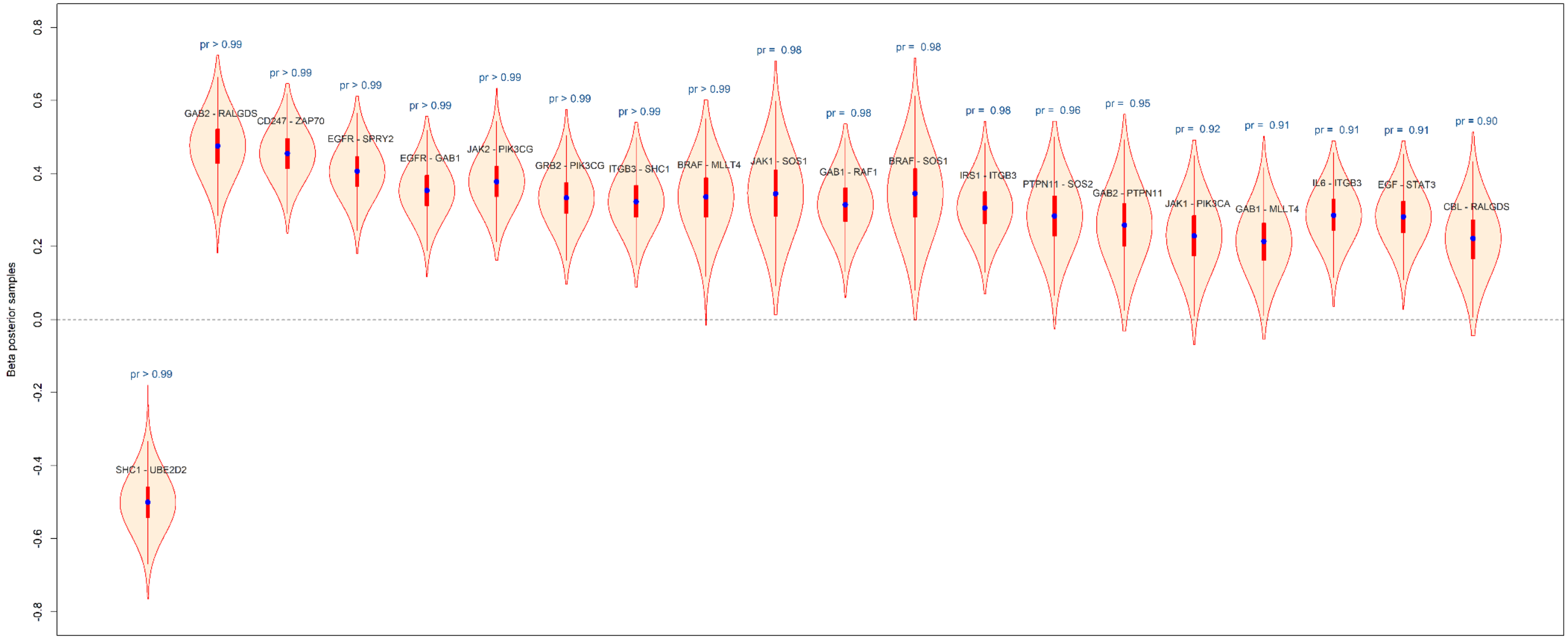

Table S6. Details about the 20 edges (existence probability > 0.9).

(1) Gene1, Gene2: the two genes connected by the selected edge

(2) Prob(edge): the estimated probability of existence

(3) Beta.mean: the posterior mean

(4) Beta.SD: the standard deviation of the beta posterior samples

(5) Q2.5%, Q97.5%, and C.I. width: the quantiles and width of the 95% credible interval of beta

Gene1	Gene2	Prob(edge)	Beta.Mean	Beta.SD	Q2.5%	Q97.5%	C.I. width
SHC1	*UBE2D2*	>0.99	-0.50	0.06	-0.62	-0.38	0.24
GAB2	*RALGDS*	>0.99	0.47	0.07	0.33	0.61	0.27
CD247	*ZAP70*	>0.99	0.45	0.06	0.34	0.57	0.23
EGFR	*SPRY2*	>0.99	0.41	0.06	0.29	0.52	0.24
EGFR	*GAB1*	>0.99	0.35	0.06	0.23	0.47	0.23
JAK2	*PIK3CG*	>0.99	0.38	0.06	0.26	0.50	0.24
GRB2	*PIK3CG*	0.99	0.33	0.06	0.21	0.46	0.25
ITGB3	*SHC1*	0.99	0.32	0.07	0.20	0.45	0.25
BRAF	*MLLT4*	0.99	0.33	0.08	0.18	0.49	0.31
JAK1	*SOS1*	0.98	0.34	0.09	0.15	0.53	0.38
GAB1	*RAF1*	0.98	0.32	0.07	0.19	0.45	0.26
BRAF	*SOS1*	0.98	0.35	0.10	0.15	0.53	0.38
IRS1	*ITGB3*	0.98	0.31	0.06	0.18	0.43	0.25
PTPN11	*SOS2*	0.96	0.28	0.08	0.11	0.44	0.33
GAB2	*PTPN11*	0.95	0.26	0.09	0.08	0.43	0.34
JAK1	*PIK3CA*	0.92	0.23	0.08	0.06	0.38	0.32
GAB1	*MLLT4*	0.91	0.21	0.07	0.07	0.36	0.29
IL6	*ITGB3*	0.91	0.28	0.06	0.16	0.40	0.25
EGF	*STAT3*	0.91	0.28	0.06	0.15	0.40	0.25
CBL	*RALGDS*	0.90	0.22	0.08	0.05	0.37	0.32

Table S7. The top three hub nodes in each method's estimated network. In the first column, the value in the parentheses represents number of edges in the estimated network by each method. The value in the parentheses in columns 2 ~ 4 denotes the number of edges connected with hub genes in the network. BMRF.P with the different selected thresholds (0.5, 0.7, 0.9) are shown in the first three rows.

	Hub node1	Hub nodes 2	Hub node 3
BMRF.P_05 (55)	*CBL* (9)	*SOS1* (8)	*PTPN11* (7)
BMRF.P_07 (41)	*CBL* (6)	*PTPN11* (6)	*JAK1* (5)
BMRF.P_09 (20)	*GAB1* (3)	*ITGB3* (3)	*EGFR* (2)
M&B (61)	*CBL* (9)	*SOS1* (9)	*PTPN11* (8)
Glasso (100)	*CBL* (13)	*SOS1* (13)	*GAB1* (12)
SPACE (57)	*CBL* (10)	*SOS1* (9)	*PTPN11* (7)
CLIME (60)	*SOS1* (11)	*CBL* (10)	*JAK1* (9)
BD_BMA (70)	*GAB1* (8)	*JAK1* (8)	*STAT3* (8)

Table S8. Numbers of identified edges. Numbers in the diagonal represent the number of edges identified by each method. Numbers in the upper triangle represent the number of edges identified by both the column and row methods. Numbers in the lower triangle represent the number of edges identified by the column method but not the row method.

	BMRF.P	SPACE	CLIME	M&B	BD_BMA	Glasso
BMRF.P	55	55	42	54	44	55
SPACE	0	57	42	55	44	56
CLIME	13	15	60	43	41	52
M&B	1	2	17	61	46	60
BD_BMA	11	13	19	15	70	49
Glasso	0	1	8	1	21	100
